# Supplementary material for: Widespread AAV-mediated CSPG digestion impairs functional recovery after cervical spinal cord injury
Source: bioRxiv. 2026 May 26:2026.05.22.727165. Preprint. [Version 1] doi: 10.64898/2026.05.22.727165 (PMC13232321; doi:10.64898/2026.05.22.727165)
Supplement: Supplement 1 [file NIHPP2026.05.22.727165v1-supplement-1.pdf]

bioRxiv preprint doi: https://doi.org/10.64898/2026.05.22.727165; this version posted May 26, 2026. The copyright holder for this preprint (which was not certified by peer review) is the author/funder, who has granted bioRxiv a license to display the preprint in perpetuity. It is made available under a CC-BY 4.0 International license.

# SUPPLEMENTARY FIGURES

**Supplemental Figure 1**

**AAV-PGK-chABC construct**

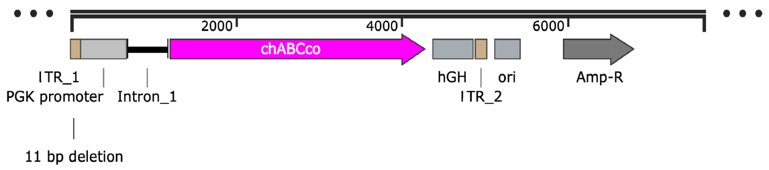


**Supplementary Figure 1.** ***Schematic of the AAV expression cassette encoding codon-optimized chABC.*** Linear schematic of the AAV2 vector used in this study. The codon-optimized chABC sequence was cloned into a compact expression cassette under the control of the PGK promoter and followed by a human growth hormone (hGH) polyadenylation signal. An intron upstream of the coding sequence and flanking LTR elements are indicated, including an 11 bp deletion within the LTR. Additional plasmid backbone elements (origin of replication, ori; ampicillin resistance, AmpR) are shown for reference. Total construct length is 7,639 bp.

bioRxiv preprint doi: https://doi.org/10.64898/2026.05.22.727165; this version posted May 26, 2026. The copyright holder for this preprint (which was not certified by peer review) is the author/funder, who has granted bioRxiv a license to display the preprint in perpetuity. It is made available under a CC-BY 4.0 International license.

**Supplemental Figure 2**

**Table 1:** **Time-resolved post hoc comparissons in the staircase reaching task**

| Time (dpi) | 7 | 11 | 14 | 18 | 21 | 25 | 30 | 35 | 39 | 42 | 44 |
|---|---|---|---|---|---|---|---|---|---|---|---|
| GFP vs chABC | | | | * | * | | | | | * | * |
| GFP vs Combo | | | ** | * | ** | * | * | ** | * | * | ** |

**Supplementary Figure 2.** ***Time-resolved post hoc comparisons of staircase reaching performance following AAV-mediated interventions.*** Post hoc comparisons were performed using a mixed-effects model with Dunnett's multiple comparisons test to evaluate differences between treatment groups at each time point. No significant differences were observed at early time points (7-11 dpi). Beginning at 14 dpi, the combination group exhibited reduced performance compared to GFP controls. At later time points (18-44 dpi), both AAV2-chABC and combination-treated animals showed reduced performance relative to GFP controls, with more consistent effects in the combination group. Symbols indicate significant differences relative to GFP controls (Dunnett's test; *$p < 0.05$, **$p < 0.01$, ***$p < 0.001$).
